# The future of periodic limb movements during sleep: beyond the periodic limb movement index

**DOI:** 10.1093/sleep/zsag121

**Published:** 2026-04-28

**Authors:** Lourdes M DelRosso

**Affiliations:** Department of Medicine, University of California, San Francisco, Fresno, CA, United States

Periodic limb movements during sleep (PLMS) have long occupied an uncertain place in sleep medicine: common enough to be routinely encountered, measurable enough to appear objective, yet still incompletely understood in their pathophysiology and clinical significance [1–4]. For decades, the field has relied heavily on the periodic limb movement index (PLMI), a convenient numerical summary that counts events per hour of sleep. The PLMI, however, does not capture all the clinical dimensions of these movements, including laterality and asymmetry [5], appearance during wakefulness [6], periodicity versus non-periodicity [7], night-to-night variability [8], and coupling with arousals or respiratory events [2]. A single index can’t capture the full picture, as evidenced by the lack of agreement between diverse scoring criteria [9, 10].

The International PLMS Taskforce roadmap is, therefore, both timely and necessary. By emphasizing harmonized scoring, multidimensional phenotypes, wearable technologies, longitudinal cohorts, and translational science, the Taskforce recognizes that the next era of PLMS research must move beyond the index, toward understanding phenotype mechanisms and clinical consequences [11].

Evidence for the need to move beyond the PLMI is already visible in population data. Recent clinical data from a diverse US cohort found higher PLMI with advancing age, male sex, and self-reported White race, while Black participants demonstrated significantly lower PLMI after adjustment for covariates [12]. Similar findings have emerged from community cohorts in which Black adults and Chinese–American participants had a lower prevalence of PLMS or combined restless legs syndrome (RLS)-PLMS phenotypes than White participants [13]. In Brazil, individuals with African ancestry also showed lower odds of PLMS than those of European ancestry [14]. Studies from Japan and Korea likewise suggest lower PLMS positivity rates among Asian patients with RLS than in many Western cohorts, although advancing age and male sex remain consistent correlates [15, 16]. In children, White participants were more likely than African American peers to demonstrate PLMS, even while the latter carried greater burdens of sleep-disordered breathing [17].

Furthermore, PLMS often do not occur in isolation but arise in the context of various comorbidities, such as renal disease [18], cardiovascular disease [19, 20], diabetes [21], narcolepsy [22], RLS [23], medication effects [24], respiratory events [25], neuropathy [26], circadian disruption, and developmental neurobiology [27]. The clinically consequential phenotype may not be defined by PLMI alone, but by combinations of autonomic activation, arousal burden, timing, comorbidity, age, sex, ancestry, or underlying neurologic susceptibility. In fact, when all these considerations are taken into account, not all PLMS will be biologically equivalent, as emerging data reveal.

Other challenges in PLMS research with direct clinical implications are the marked heterogeneity in sleep-related limb movements observed across the lifespan. Children frequently present not with textbook periodic limb movements, but with isolated, aperiodic, or mixed lower-extremity movements associated with restless sleep, behavioral dysregulation, or daytime impairment. A recent proposal from the International Restless Legs Study Group reconceptualizes pediatric periodic limb movement disorder within a broader category of sleep leg movement disorder of childhood, acknowledging this reality and challenging the assumption that adult periodicity rules should define pediatric limb movements [26]. A developmental perspective may be especially important, as iron deficiency in infancy has been associated with persistent alterations in later sleep-motor regulation, manifested as periodic limb movements [28]. Given iron’s central role in dopamine metabolism, oligodendrocyte function, and myelination of descending motor pathways, some adult PLMS phenotypes may reflect the delayed expression of earlier neurodevelopmental vulnerability. Because most individuals undergo sleep testing only after symptoms such as snoring or disrupted sleep emerge, longitudinal population studies are essential to define the development, natural history, and true global prevalence of PLMS.

Beyond these observations lie more fundamental scientific questions. Do current datasets and scoring frameworks adequately represent global populations in the identification of PLMS and related limb movements? Do ancestry-related biologic factors influence motor periodicity, iron handling, or dopaminergic signaling? Are environmental stressors, sleep fragmentation, comorbidity burden, medication exposure, or access to care shaping what is captured as PLMS in polysomnography? The most likely answer is that each contributes in part. Diversity, therefore, should be viewed as a scientific opportunity to refine the pathophysiological understanding of PLMS.

Genetics provides an important framework for refining our understanding of PLMS heterogeneity. Genome-wide association studies have identified susceptibility loci implicated in RLS, including MEIS1 and BTBD9, supporting shared pathways involving neurodevelopment, sensorimotor regulation, and sleep-related motor control [29, 30]. PLMS also demonstrates weaker genetic correlations with insomnia and a possible association with stroke or cardiovascular risk [31]. Recent studies further underscore a crucial distinction between spontaneous PLMS and respiratory-related leg movements (RRLMs), which occur in the setting of obstructive sleep apnea. Emerging evidence suggests that RRLMs may involve distinct pathophysiologic mechanisms, driven more by arousal and autonomic surges than by the sensorimotor pathways classically implicated in RLS, and have been independently associated with increased all-cause mortality, whereas the traditional PLMI was not. Longer respiratory-related leg movements were also linked to more severe respiratory events, greater oxygen desaturation, arousals, and larger heart rate responses [32].

Taken together, these challenges also define the opportunities ahead. Emerging analytic platforms, wearable sensors, and machine learning tools offer tremendous promise for scalable PLMS detection and multidimensional phenotyping. Standardized automated scoring may reduce interscorer variability, improve efficiency, and expand access to evaluation and treatment beyond specialized sleep centers. Yet algorithms inevitably inherit the strengths and weaknesses of the datasets used to train them. If training cohorts underrepresent racial minorities, women, medically complex older adults, children, or underserved populations, automated systems may codify incomplete definitions of normality and pathology.

The PLMS Taskforce appropriately calls for a reimagining of the field [11]. As advances in automated scoring, wearable technologies, and large-scale datasets reshape PLMS research, future progress must also address the needs of regions where access to polysomnography and advanced technologies remains limited. The most meaningful innovations will be those that are accurate, affordable, and applicable across diverse global settings, while integrating clinical insight and perspectives across ages, backgrounds, and communities. Only then will PLMS evolve from a numerical polysomnographic index into a clinically meaningful biomarker capable of advancing sleep health and improving patient outcomes.

## Disclosure statement

*Financial disclosure*: None declared.

*Non-financial disclosure*: The author does not have any conflict of interest or financial interest to declare.
